# Supplementary material for: Cooperative agreement between countries of the North Atlantic Ocean reduces marine plastic pollution but with unequal economic benefits
Source: Commun Earth Environ. 2025 Feb 22;6(1):134. doi: 10.1038/s43247-025-02115-5 (PMC11845319; doi:10.1038/s43247-025-02115-5)
Supplement: Supplementary file 2 — Supplementary Information [file 43247_2025_2115_MOESM2_ESM.pdf]

Supplementary Information for

"Cooperative agreement between countries of the North Atlantic Ocean reduces marine plastic pollution but with unequal economic benefits"

Nicola J. Beaumont[1], Tobias Börger[2], James R. Clark[1], Nick Hanley[3], Robert J. Johnston[4], Keila Meginnis[5], Christopher Stapenhurst[6], Frans P. de Vries[7*]

[1] Plymouth Marine Laboratory, Plymouth, England, UK

[2] Berlin School of Economics and Law (HWR Berlin), Badensche Straße 52, 10825 Berlin, Germany

[3] School of Biodiversity, One Health and Veterinary Medicine, University of Glasgow, Scotland, UK

[4] George Perkins Marsh Institute, Clark University, Worcester, MA, USA

[5] Patient-Centered Research, Evidera; School of Biodiversity, One Health and Veterinary Medicine, University of Glasgow, Scotland, UK

[6] Quantitative Social and Management Sciences Research Centre (QSMS), Faculty of Economics and Social Sciences, Budapest University of Technology and Economics, Budapest, Hungary

[7] Department of Economics, Business School, University of Aberdeen, Scotland, UK

[*]Corresponding author: Frans de Vries (frans.devries@abdn.ac.uk)

Including:

Supplementary Notes 1 to 7

Supplementary Figures 1 to 5

Supplementary Tables 1 to 6

Supplementary Notes 1. Choice of countries and territories

We limited our study to a selection of countries surrounding the North Atlantic Ocean, where the North Atlantic and surrounding coastal waters play a dominant role in facilitating the transboundary transfer of marine plastic debris. The study was further limited to countries for which consistent plastic river emissions data was available[1]. The southern boundary of the study was drawn at Mexico and Morocco on the Western and Eastern sides of the North Atlantic respectively. Given the highly restricted flow of water between the North Atlantic and the Mediterranean Sea through the Strait of Gibraltar, we excluded all Mediterranean countries without a coastline facing onto the North Atlantic. Another notable omission was Norway, for which we lacked consistent data on plastic emissions. Of the complex set of small island states and overseas territories in the Antilles, only Haiti and the Dominican Republic were included. Cuba, which is the largest island in the area, was excluded as we lacked plastic emissions data for it. Lastly, in North Africa, the disputed territory of Western Sahara was joined with Morocco and included as a single territory for the purpose of the study. In all cases, data on country boundaries and names were taken from the Natural Earth dataset (https://www.naturalearthdata.com/).

To determine the presence and absence of plastic within the territorial waters of a given country, we defined the territorial waters of each country by its Exclusive Economic Zone (EEZ). The boundary for each EEZ was taken from v11 of the Marine Boundaries dataset (https://www.marineregions.org)[2]. Within the Marine Boundaries dataset, the boundary of EEZs for some exclave and semi-exclave regions are entered separately. With a few exceptions, these were combined to give the full EEZ of the associated country (Supplementary Table 1). Some exclave regions were excluded. In the case of France, we limited the study to European France, including the island of Corsica; other regions of France, including French Guiana in South America, were excluded. Although we decided to include the Pacific facing US states of Alaska and Hawaii, their contribution to inter-country transfers of plastic was small. If a region was excluded, both its emissions and its associated EEZ were omitted from the study.

Supplementary Notes 2. Particle release scenarios

The location of all river mouths was extracted from the Meijer et al (2021) dataset[1]. The Meijer dataset was chosen as it dealt explicitly with buoyant macroplastic and was calibrated against a wider set of observations when compared with earlier datasets[2]. River locations were associated with countries by searching for the nearest country to the river's geographic coordinates using country-level shapefile data from the Natural Earth dataset. The dataset was then trimmed so it only included rivers specific to countries listed in the study. The study was simplified in the sense that emissions from a given river were, in their entirety, associated with a single country, determined by the location of the river mouth, irrespective of whether the river's drainage basin spanned the territory of multiple countries or not.

As the 1/12° ocean model grid on which the sea surface velocity field is defined provides a relatively poor fit to the global coastline, the exact location around which particles were released into the model domain were determined by searching for the centroid of the nearest ocean element to the river's mouth. These locations were then used as the central coordinates for a set a circular release zones, each of radius 1 km, and within which 100 particles were randomly scattered. In the Meijer et al dataset[1], there were 3217 rivers associated with the 16 countries included in the study, yielding a total of 3.217 x 10^5 particles per simulation.

The Meijer et al dataset[1] lists the amount of plastic emitted by each river per annum. In our simulations, we spread the emissions over a year by releasing one set of particles per month, resulting in 12 particle releases per year. The decision represents a balance between capturing short term variations in currents and overall computational complexity. In all cases, particles were released at 1200 on the 1st of each month and their positions saved to file at 1200 on each day of the simulation. In the set of core simulations, monthly particle releases were started at 1200 on 1st January 2000 and ended with a particle release at 1200 on 31st December 2014. Each monthly release was run as a separate simulation, yielding 180 simulations involving more than 5.0 x 10^7 particles in total. All simulations were terminated at 1200 on 1st January 2015. A second set of slightly longer runs were performed to investigate the sensitivity of the model results to parameter choices and different configuration options. In these simulations, a set of 12 monthly releases were performed for the year 1995. In all cases, the model runs were again terminated at 1200 on 1st January 2015.

Supplementary Notes 3. Calculation of plastic inventories and stocks

The total mass of plastic at time t, $M(t)$, from a single monthly release is calculated using

$$M(t) = \sum_{i=1}^{N_\mathrm{P}} \sum_{j=1}^{N_\mathrm{R}} w_{ij}(t) \,, \tag{A1}$$

where N_R is the number of rivers. The total amount of plastic in the ocean, M_T, resulting from an ensemble of releases, is found by further summing equation (A1) over the set of monthly releases, while the contribution of an individual country to the total inventory is found by limiting the set of rivers, N_R, to rivers from that country alone. The stock of plastic in a country's EEZ is computed by summing the masses of just those particles that lie within the EEZ at time t (see also section Supplementary Notes 4).

Supplementary Notes 4. Calculation of plastic fluxes between countries

The flux of plastic moving between countries is calculated based on the presence-absence of plastic within the EEZs of each country, as determined from particle positions which are defined at discrete points in time. The flux of plastic, τ_{kl}, between two countries k and l, is defined as the annual average daily flux of plastic that flows between the two countries. It is used to compute the export fraction, as showing in Figure 1c of the main article. For a single simulation in which particles are released at time $t = t^0$, τ_{kl} is calculated using

$$\tau_{kl} = \frac{1}{N_\mathrm{e} - N_\mathrm{s}} \sum_{n=N_\mathrm{s}}^{N_\mathrm{e}} \sum_{i=1}^{N_{\mathrm{P_T}}} w_i^{n+1} \, I_{i,k+}^{n} I_{i,l+}^{n+1},$$

(A2)

where $N_\mathrm{s}(t^0)$ and $N_\mathrm{e}(t^0)$ correspond, respectively, to the first and last day indices of the target year for which fluxes are being calculated; $N_{\mathrm{P_T}}$ is the total number of particles released from all rivers in all countries; and $I_{i,k+}^{n}$ and $I_{i,l+}^{n+1}$ are discrete variables calculated using case expressions of the form

$$I_{i,k+}^{n} = \begin{cases} 1, & \text{if particle } i \text{ is in the EEZ of country } k \text{ at time point } n \\ 0, & \text{otherwise} \end{cases},$$

which identify whether a particle resides within the EEZ of a country or not – as determined by testing whether the particle's point location lies within a polygon (or polygons) that defines the area of the EEZ. Fluxes are calculated using particle weights defined at the time point, $n + 1$, reflecting the mass of plastic in the EEZ of receiving country l which was previously in the EEZ of country k. The difference between the particle's weight at the two time points, $\Delta w = w^n - w^{n+1}$, is the fraction which is assumed to have sunk away from the sea surface, or to have been removed by other processes not explicitly represented in the model, as determined by each particle's decay factor. It should be noted that in this method, no special attention is given to where the plastic originally came from (i.e., the river and country from which it was emitted).

In the set of core simulations, monthly releases were repeated at 1200 on the first day of each month, starting at 1200 on 1st January 2000. While setting t^0 equal to the date/time of each monthly particle release, a single set of fluxes for all members of the ensemble was formed by summing the fluxes corresponding to each monthly release (equation (A2)) for the given target year.

Supplementary Notes 5. Calculation of exposure to plastic waste originating from different countries

A second metric called exposure was introduced to further investigate and visualise how each country was impacted by the emissions from other countries; and to explore model sensitivity. The exposure metric, H_{kl}, is designed to account for both the mass of surface plastic within the EEZ of country *l*, and the amount of time it spends within with the EEZ, following its emission by country *k*. The metric is effectively an integral over time, which accounts for changes in the weights of particles in time. Exposure from a single monthly release of particles is calculated from particle positions and weights using

$$H_{kl} = \sum_{n=1}^{N_e} \sum_{i=1}^{N_{P_k}} w_i^n \, I_{i,l+}^n,$$

(A3)

where the summation is performed for just those particles, N_{P_k}, emitted from rivers associated with country *k*. Here, the summation in time is done over all time points from the start of the simulation to some end time index, N_e. Exposure from cumulative monthly particle releases, performed over a single year, were calculated by further summing equation (A3) over the set of monthly releases.

Supplementary Notes 6. Choice modelling methodology

Discrete choice experiment surveys were conducted in two treatments: in the UK (with the UK referred to as "home" and the US as "foreign" country) and US (where the US was the home and the UK the foreign country). The development and application of the discrete choice experiment are described in Börger et al[4]. The specific model to analyse these data differs from that in the paper and is explained in the following. The choice data were analysed using a mixed multinomial logit model in WTP-space[5,6]. In this model, the utility of respondent n choosing alternative i in choice occasion t, $U_{nit}(\boldsymbol{X_{nit}}, C_{nit})$, is assumed to consist of an observable component $V_{nit}(\boldsymbol{X_{nit}}, C_{nit})$ and an error term ε_{nit}

$$U_{nit}(\boldsymbol{X_{nit}}, C_{nit}) = V_{nit}(\boldsymbol{X_{nit}}, C_{nit}) + \varepsilon_{nit} = \boldsymbol{\beta_n'} \boldsymbol{X_{nit}} - \gamma_n C_{nit} + \varepsilon_{nit}.$$

(A4)

It is further assumed that $V_{nit}(\cdot)$ can be represented by a linear-additive indirect utility function consisting of the matrix $\boldsymbol{X_{nit}}$ containing the values of all non-monetary attributes of i; C_{nit}, the cost to the decision-maker of i; as well as $\boldsymbol{\beta_n}$ and γ_n, a conforming parameter vector and scalar to be estimated. The above model in preference space can be transformed into WTP-space as

$$V_{nit}(\boldsymbol{X_{nit}}, C_{nit}) + \varepsilon_{nit} = \gamma_n(\boldsymbol{\delta_n} \boldsymbol{X_{nit}} - C_{nit}) + \varepsilon_{nit} \,,$$

(A5)

where $\boldsymbol{\delta_n} = \boldsymbol{\beta_n}/\gamma_n$. While equations (A4) and (A5) are behaviourally equivalent, the elements of $\boldsymbol{\delta_n}$ in (5) can be interpreted directly as marginal WTP estimates for the changes in the different attributes. To

accommodate possible preference heterogeneity, all parameters are subscripted by n to allow them to vary over decision-makers. In the model in WTP-space, assumptions about the form of the distribution of the parameters can be made directly for the elements of $\boldsymbol{\delta_n}$ and γ_n; the former of which are assumed to follow a normal distribution, the latter of which follows a lognormal distribution with sign change.

Assuming that ε_{nit} follows a Type I Extreme Value distribution and that respondent n in each choice situation $t = 1, \dots, T$ selects the alternative which maximises their utility, the joint probability of a series of choices $\boldsymbol{y_n} = \langle y_{nt}, \dots, y_{nT} \rangle$ is

$$Pr(\boldsymbol{y_n} | \boldsymbol{X_n}, \boldsymbol{C_n}) = \prod_{t=1}^{T} \frac{e^{V_{nit}}}{\sum_{j=1}^{J} e^{V_{njt}}} \; . \tag{A6}$$

Since the mixed multinomial logit model does not have a closed-form solution, simulated maximum likelihood is used to estimate the parameters in equation (A5). 1,000 Sobol draws are used to simulate the likelihood function. Models are estimated in R[7] using the ‘Apollo’ package[8,9]. To promote convergence, both the cost variable C_{nit} and the percentage changes in plastic pollution in $\boldsymbol{X_{nit}}$ are divided by 100 prior to estimation. Hence, the resulting WTP estimates ($\boldsymbol{\delta'_n}$) are directly interpretable as marginal WTP estimates (in US dollars) for a one-percent change in pollution.

Importantly, in this specification, the level of attribute k in one specific choice alternative is calculated based on the percentage change in area-specific plastic pollution, A, as $x_{nit}^{k} = A\left(1 - \frac{A}{2}\right)$. With this specification, the estimated parameter δ^k can be interpreted as the marginal (money-metric) utility of changing the level of pollution by one percentage point while ensuring that the marginal utility is equal to δ^k when no abatement happens (i.e., $A = 0$) and $0.5\delta^k$ at full abatement (i.e., $A = 1$). The results of the two mixed multinomial logit models are presented in Supplementary Table 6.

Supplementary Notes 7. Sensitivity analysis calculations

Define $Q_i := \sum_{l\in N} T_{li}$ as the quantity of MPP flowing into country i's waters. We use the product and quotient rules to calculate

$$\begin{aligned}\partial S_{ii}/\partial T_{jk} &= \mathcal{I}(k=i)(\mathcal{I}(j=i)Q_i - T_{ii})/Q_i^2 \\ &= \mathcal{I}(k=i)(\mathcal{I}(j=i) - S_{ii})/Q_i\end{aligned}$$

$$\begin{aligned}\partial R_i(a)/\partial T_{jk} &= \mathcal{I}(k=i)\left(a_k Q_i - \sum_{j\in N} a_j T_{ji}\right)/Q_i^2 \\ &= \mathcal{I}(k=i)\big(a_k - R_i(a)\big)/Q_i\end{aligned}$$

$$\begin{aligned}\partial \beta_j/\partial y_i &= \left(\mathcal{I}(i=j)\epsilon y_j^{\epsilon-1}\sum_k y_k^\epsilon - y_j^\epsilon \epsilon y_i^{\epsilon-1}\right)/\left(\sum_k y_k^\epsilon\right)^2 \\ &= \epsilon\big(\mathcal{I}(i=j)y_j^{\epsilon-1} - \beta_j y_i^{\epsilon-1}\big)/\sum_k y_k^\epsilon \\ &= \beta_j\epsilon\big(\mathcal{I}(i=j)/y_j - \beta_i/y_i\big)\end{aligned}$$

$$\begin{aligned}\partial \beta_i/\partial \epsilon &= \left[\ln(y_i)y_i^\epsilon\left(\sum_{j\in N} y_j^\epsilon\right) - y_i^\epsilon \sum_{j\in N}\ln\big(y_j\big)y_j^\epsilon\right]/\left(\sum_{j\in N} y_j^\epsilon\right)^2 \\ &= \left[\ln(y_i)y_i^\epsilon - \beta_i\sum_{j\in N}\ln\big(y_j\big)y_j^\epsilon\right]/\sum_{j\in N} y_j^\epsilon \\ &= \beta_i\left(\ln(y_i) - \sum_{j\in N}\ln\big(y_j\big)\beta_j\right).\end{aligned}$$

Then the chain rule gives

$$\begin{aligned}\partial V(a;\theta)/\partial T_{jk} &= \sum_{i\in N}\mathcal{I}\,(k=i)\beta_i\big[\big(a_i - R_i(a)\big)\big(2 - R_i(a)\big) - R_i(a)a_j + 2(\mathcal{I}(j=i) - S_{ii})\ln(1-a_i)/Q_i\big] \\ &= 2\beta_k\big[\big(a_k - R_k(a)\big)\big(1 - R_k(a)\big) + (\mathcal{I}(j=k) - S_{kk})\ln(1-a_k)/Q_i\big],\end{aligned}$$

$$\begin{aligned}\partial V(a;\theta)/\partial y_i &= \epsilon\sum_{j\in N}\big(\mathcal{I}(i=j)/y_j - \beta_i/y_i\big)\,v_j(a) \\ &= \epsilon v_i(a)/y_i - \beta_i/y_i\sum_{j\in N} v_j\,(a) \\ &= \epsilon(v_i(a) - \beta_i V)/y_i,\end{aligned}$$

and

$$\begin{aligned}\partial V(a;\theta)/\partial \epsilon &= \sum_{i\in N}\left(\ln(y_i) - \sum_{j\in N}\ln\big(y_j\big)\beta_j\right)v_i(a) \\ &= \sum_{i\in N}\ln\,(y_i)v_i(a) - \sum_{j\in N}\ln\big(y_j\big)\beta_j V \\ &= V\sum_{i\in N}\ln\,(y_i)(v_i(a)/V - \beta_i).\end{aligned}$$

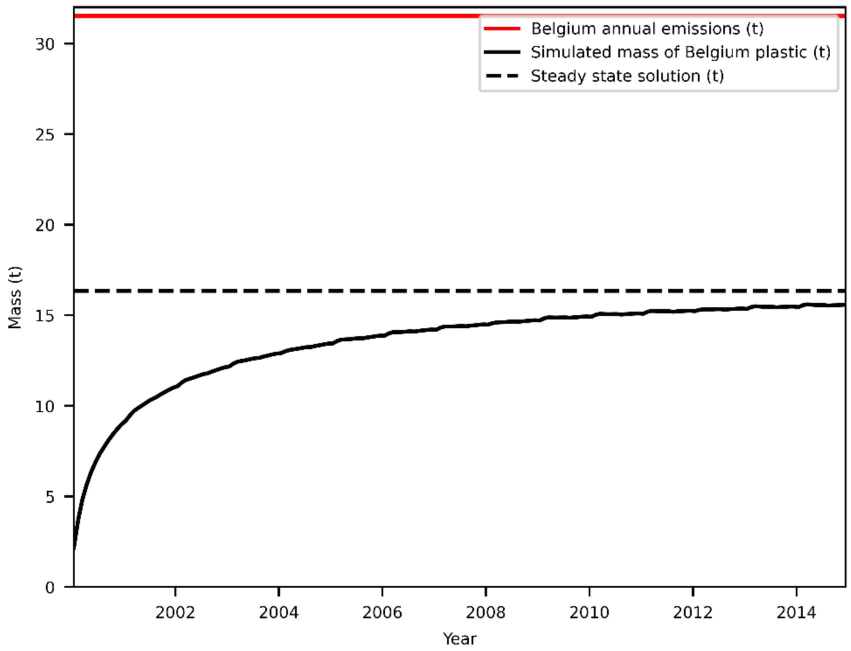

Supplementary Figure 1. The accumulation of surface plastic emitted by a single country (Belgium) as a function of time. With constant emissions, the simulated inventory of surface plastic asymptotes toward an imposed steady state where new inputs are balanced by losses from the surface. Results from the model are analysed in the years 2012-2014.

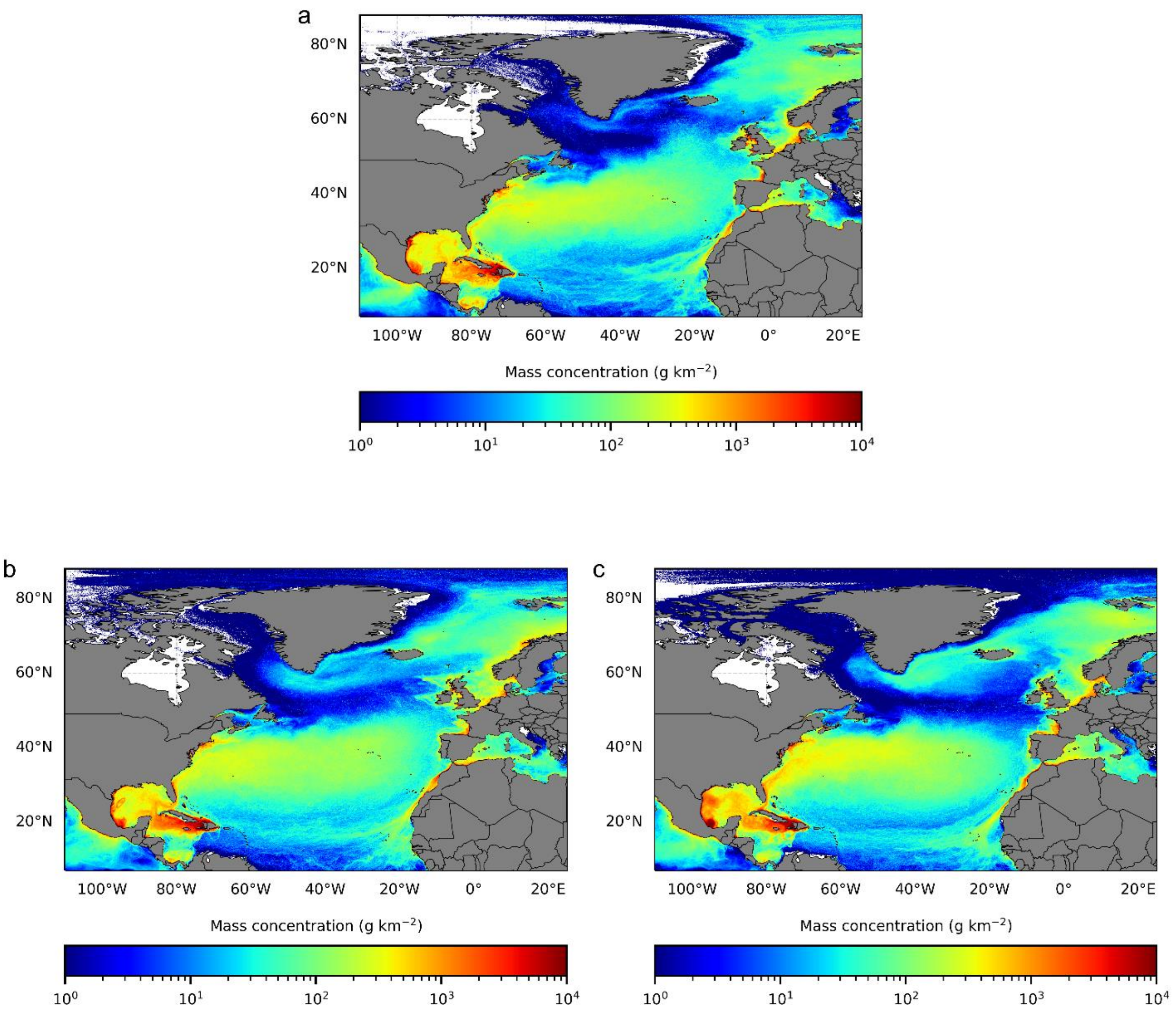

Supplementary Figure 2. Variations in the annual mean mass concentration of plastic in the years 2012 (a), 2013 (b) and 2014 (c).

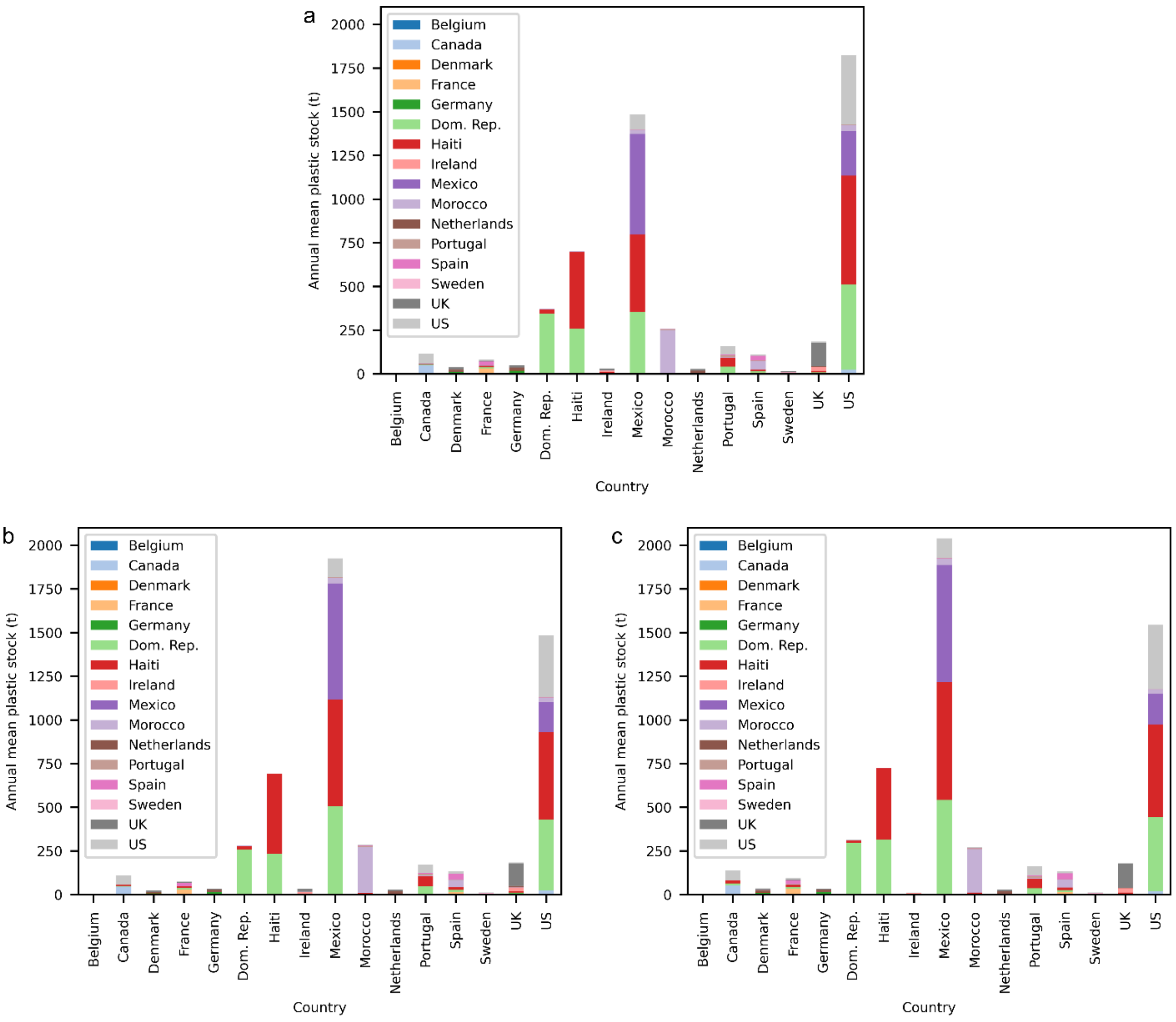

Supplementary Figure 3. Variations in the annual mean plastic stock within the EEZ of each country in the years 2012 (a), 2013 (b) and 2014 (c); with colours indicating the country from which the plastic originated.

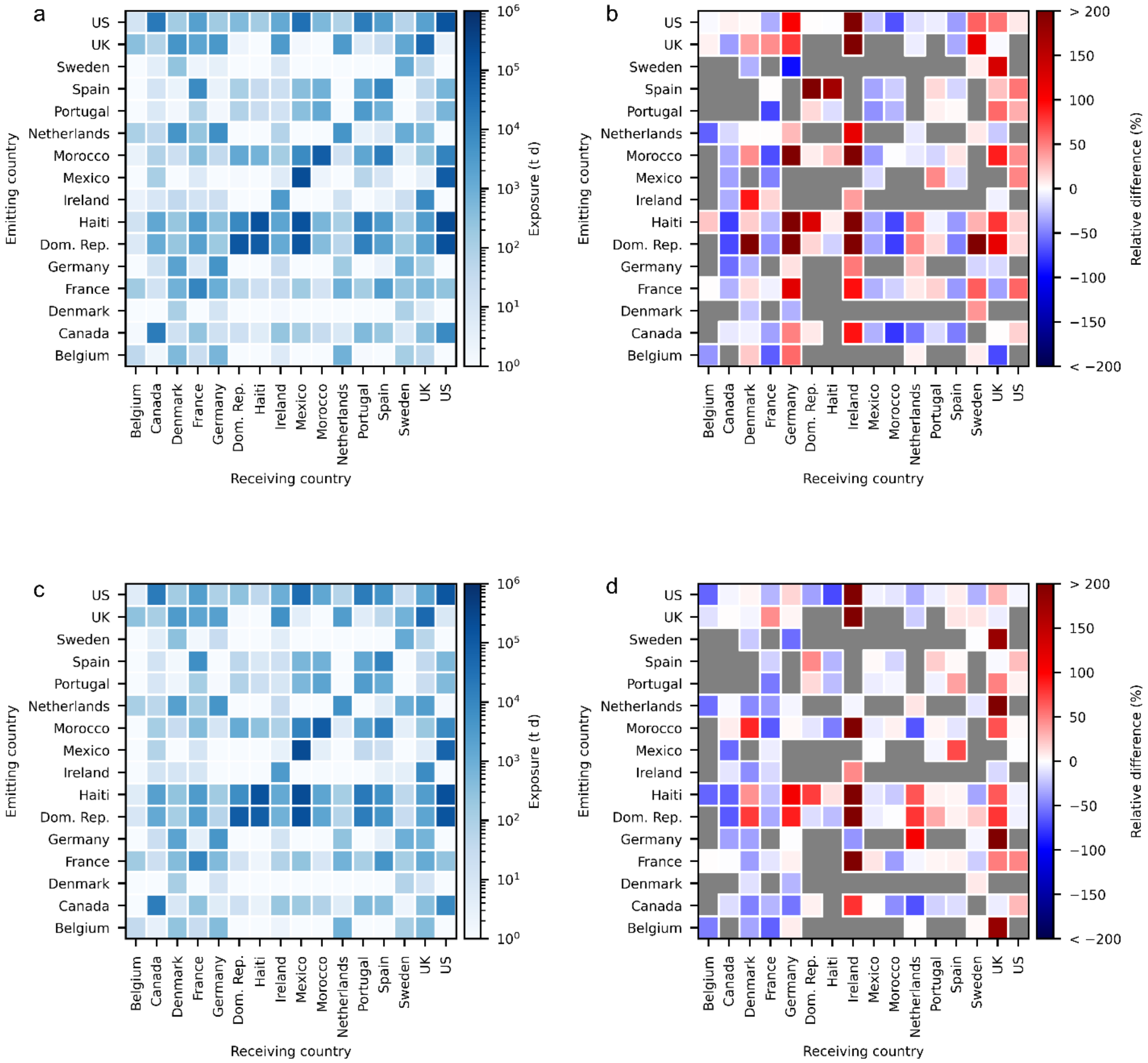

Supplementary Figure 4. Transfer matrices showing exposure assessed in the years 2012 (a) and 2013 (c), and the difference relative to exposure assessed in the year 2014 (b and d, respectively). Receiving countries with exposures < 10 t d, based on a given country's emissions, have been masked (grey shading in figures b and d). The calculation of exposure is described in section Supplementary Notes 5 of the SI.

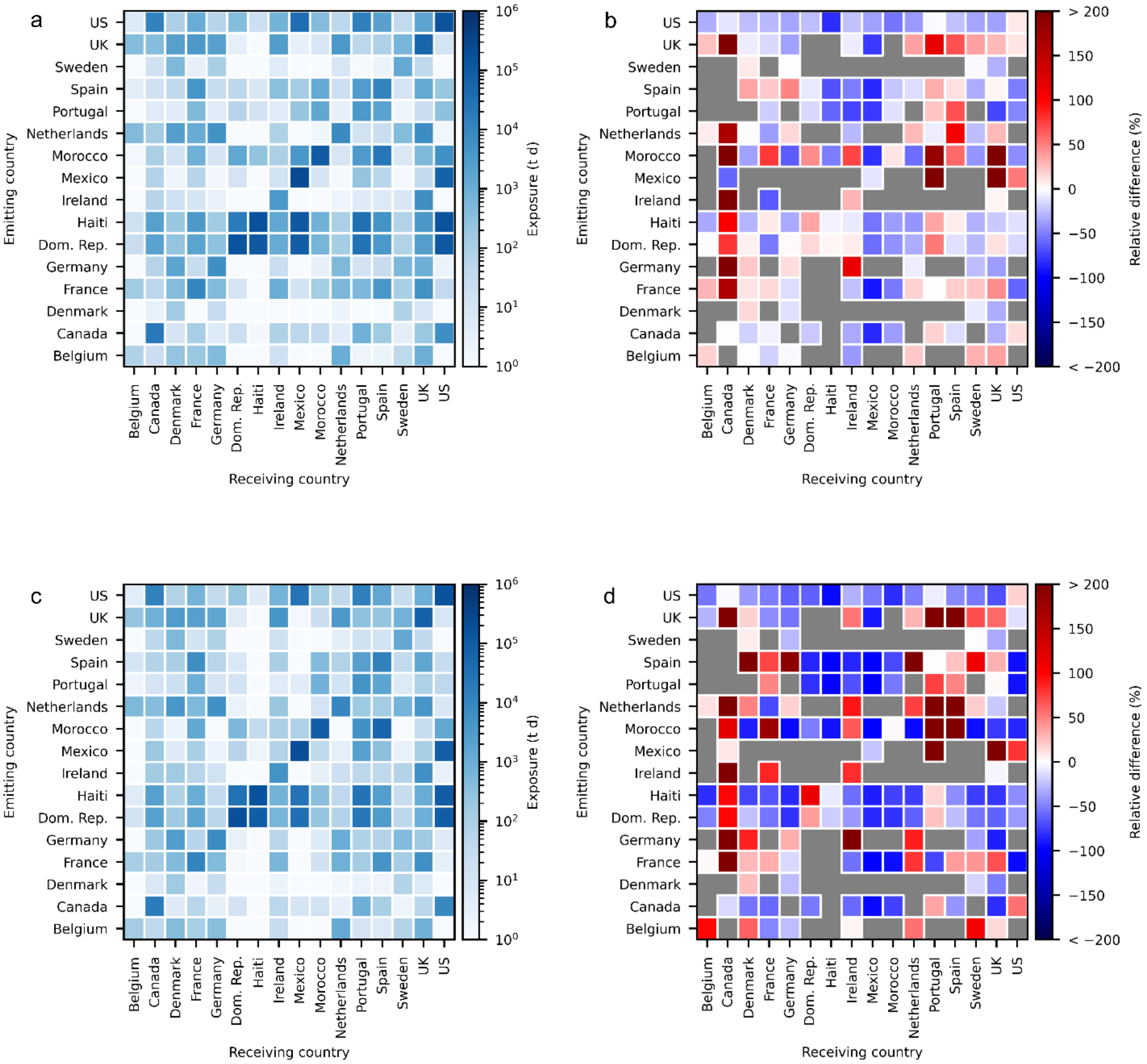

Supplementary Figure 5. Difference plots showing the percentage change in exposure for different values of the wind factor. Exposure is calculated for a single year of emissions, in which plastic was released on the 1st of each month in the year 1995. Exposure is calculated over a period that extends from 1st January 1995 – 31st December 2014. a) Exposure calculated using a model of leeway and using a wind factor of 1% in the direction of the wind. b) Percentage difference in exposure for two runs using wind factors of 1% and 2%. c) Exposure calculated using a wind factor of 0% (i.e., particles are moved by surface ocean currents only). d) Percentage difference in exposure for two runs using wind factors of 0% and 2%. In b) and d), exposures < 10 t d have been masked (grey cells).

Supplementary Table 1. List of countries and associated Exclusive Economic Zones (EEZs) included in the study, and their river plastic emissions as reported by Meijer et al[1].

**Country name**		**Country code**	**Exclusive Economic Zones (EEZs)**	**Number of rivers**	**Total plastic emissions / t yr^{-1}**
Belgium		BE	Belgian EEZ	8	32
Canada		CA	Canadian EEZ	241	237
Denmark		DK	Danish EEZ	22	5
Dominican Republic (Dom. Rep.)		DE	Dominican Republic EEZ	178	6027
France		DO	French EEZ	200	234
Germany		FR	German EEZ	67	132
Haiti		HT	Haitian EEZ	231	6929
Ireland		IE	Irish EEZ	80	105
Mexico		MX	Mexican EEZ	420	2218
Morocco		MA	Moroccan EEZ; Overlapping claim Western Saharan EEZ	91	1834
Netherlands		NL	Dutch EEZ	46	265
Portugal		PT	Portuguese EEZ; Portuguese EEZ (Azores); Portuguese EEZ (Madeira)	75	75
Spain		ES	Spanish EEZ; Spanish EEZ (Canary Islands)	154	214
Sweden		SE	Swedish EEZ	48	36
United Kingdom (UK)		UK	United Kingdom EEZ	416	701
United States (US)		US	United States EEZ; United States EEZ (Alaska), United States EEZ (Hawaii)	940	2429

Supplementary Table 2. Results from the fully cooperative solution using plastic transfer coefficients for the year 2014 (see Figure 3 main article).

Country code	GNI $bn	β / %	v %	a %	A %
			2014		
BE	688	1.7	0.7	73.2	38.4
CA	1976	4.5	0.2	13.6	8.2
DK	390	1.0	0.5	72.7	30.8
DO	216	7.9	4.0	3.0	32.1
FR	3499	11	6.7	-19.7	28.3
DE	4961	0.5	-2.3	93.5	92.7
HT	36	0.1	-0.5	99.5	96.7
IE	399	1.0	-0.1	50.7	41.5
MX	2501	5.7	3.3	39.0	69.2
MA	328	0.81	-0.6	57.3	57.0
NL	1110	2.61	-1.4	73.0	48.0
PT	365	0.9	0.5	64.6	46.5
ES	1939	4.5	2.0	40.3	46.4
SE	636	1.5	0.6	-5.3	19.9
UK	3327	7.5	0.9	20.0	26.4
US	23393	48.9	47.4	-42.7	55.2
Total	-	100	62.0	64.3	64.3

Notes: Country codes are listed in the main article. Purchasing power parity (PPP) Gross National Income (GNI) data is for 2021. It was downloaded in December 2022 and is subject to revision[10]. β is the benefit parameter, normalised by the WTP to abate all pollution ($59bn); v is the value from the policy, given as a percentage of $59bn; a and A are the emissions and stocks abated, expressed in percentage terms. The total stock abated excludes plastic in international waters.

Supplementary Table 3. Results from the economic optimisation model for the year 2014 under a range of political economy constraints (see Figure 4 main article).

Country code	v % (C1)	v % (C2)	v % (C3)	v % (C4)	a % (C1)	a % (C2)	a % (C3)	a % (C4)
BE	0.65	0	0.96	0.52	72.04	1.03	43.16	81.12
CA	1.61	0	1.07	0.64	1.14	0.21	43.16	18.64
DK	0.52	0	0.64	0.41	70.70	89.15	43.16	74.03
DO	4.48	0	2.29	2.58	0.24	-2.41	43.16	17.28
FR	6.25	0	3.19	5.36	0	11.83	43.16	-11.30
DE	-2.11	0	-0.21	0	92.38	0.06	43.16	10.74
HT	-0.45	0	0	0	99.35	-0.08	43.16	27.63
IE	-0.07	0	0.06	0	50.26	-0.02	43.16	38.82
MX	3.84	0	1.74	0	28.67	-0.01	43.16	55.05
MA	-0.52	0	-0.29	0	54.81	0.64	43.16	17.08
NL	-1.26	0	0.35	0	71.34	1.54	43.16	48.63
PT	0.66	0	0.55	0.14	61.04	0.44	43.16	89.00
ES	2.24	0	1.78	0.47	37.80	-0.09	43.16	52.30
SE	0.67	0	0.55	0.50	0.07	-57.21	43.16	-1.63
UK	1.03	0	-1.42	0	18.61	-0.09	43.16	27.10
US	42.44	0	19.91	16.30	0	0	43.16	11.52
Total	59.98	0	31.18	26.92	66.63	0.03	43.16	24.72

Notes: v is the value from the policy, given as a percentage of \$59bn; a is the percentage of emissions abated. Results are given for the political-economy constraints C1-C4 as described in the main article.

Supplementary Table 4. Transport elasticities for the unconstrained policy.

Country code	BE	CA	DK	DO	FR	DE	HT	IE	MX	MA	NL	PT	ES	SE	UK	US	Total
BE	-4	0	1	0	1	0	0	0	0	0	0	0	0	1	0	0	-1
CA	0	-1	0	0	0	0	0	0	0	0	0	0	0	0	0	6	6
DK	0	0	0	0	0	0	0	0	0	0	0	0	0	0	0	0	0
DO	1	0	0	-1	1	0	0	0	0	0	-1	0	0	0	0	0	0
FR	0	0	-11	0	11	0	0	0	0	0	-1	0	0	-12	0	0	-13
DE	0	1	0	1	0	-11	3	1	6	0	0	2	1	0	0	129	133
HT	0	0	0	0	0	1	-5	1	6	0	0	1	0	0	-1	158	161
IE	0	0	0	0	0	0	0	-4	0	0	0	0	0	0	-3	0	-7
MX	0	0	0	0	0	0	0	0	-16	0	0	0	0	0	0	56	39
MA	0	0	0	0	0	0	0	0	0	-5	0	0	3	0	0	7	7
NL	12	0	15	0	25	0	0	0	0	0	-47	0	0	12	0	0	17
PT	0	0	0	0	0	0	0	0	0	0	0	-1	0	0	0	0	0
ES	0	0	0	9	0	0	0	0	0	0	0	1	-10	0	0	0	0
SE	0	0	0	0	0	0	0	0	0	0	0	0	0	0	0	0	0
UK	11	0	9	1	10	0	0	8	0	0	2	0	0	6	-40	0	6
US	-6	-247	-5	-47	-3	0	0	-26	-10	-5	-3	-116	-34	-5	-11	171	-348
Total	14	-246	8	-37	45	-10	-2	-21	-14	-8	-50	-114	-40	3	-56	527	0

Supplementary Table 5. Transport elasticities for the positive abatement policy.

Country code	BE	CA	DK	DO	FR	DE	HT	IE	MX	MA	NL	PT	ES	SE	UK	US	Total
BE	-4	0	1	0	1	0	0	0	0	0	0	0	0	1	0	0	-1
CA	0	0	0	0	0	0	0	0	0	0	0	0	0	0	0	0	0
DK	0	0	0	0	0	0	0	0	0	0	0	0	0	0	0	0	0
DO	0	0	0	0	0	0	0	0	0	0	-1	0	0	0	0	0	-2
FR	0	0	0	0	0	0	0	0	0	0	0	0	0	0	0	0	0
DE	0	1	0	1	0	-11	3	1	5	0	0	2	1	0	0	2	5
HT	0	0	0	0	0	1	-5	1	5	0	0	1	0	0	-1	0	2
IE	0	0	0	0	0	0	0	-4	0	0	0	0	0	0	-3	0	-7
MX	0	0	0	0	0	0	0	0	-11	0	0	0	0	0	0	2	-9
MA	0	0	0	0	0	0	0	0	0	-4	0	0	3	0	0	0	0
NL	12	0	14	0	16	0	0	0	0	0	-45	0	0	11	0	0	9
PT	0	0	0	0	0	0	0	0	0	0	0	-1	0	0	0	0	0
ES	0	0	0	8	0	0	0	0	0	0	0	1	-10	0	0	0	-1
SE	0	0	0	0	0	0	0	0	0	0	0	0	0	0	0	0	0
UK	11	0	8	1	5	0	0	7	0	0	2	0	0	5	-38	0	3
US	0	0	0	0	0	0	0	0	1	0	0	0	0	0	-1	0	1
Total	19	2	23	11	22	-9	-2	5	-1	-3	-44	2	-5	18	-43	4	0

Supplementary Table 6. Mixed multinomial logit models in WTP-space.

	UK treatment (paired foreign country: US)		US treatment (paired foreign country: UK)	
	**Coefficient**	**s.e.**	**Coefficient**	**s.e.**
Mean of random parameters				
None	-1.330**	(0.043)	-1.591**	(0.145)
Beach	1.510**	(0.139)	2.252**	(0.169)
Coastal	1.559**	(0.265)	2.196**	(0.341)
International	1.172**	(0.203)	2.051**	(0.380)
Foreign	0.628**	(0.147)	0.541*	(0.279)
Csplit_25home	-0.091**	(0.030)	-0.327**	(0.061)
Csplit_75home	-0.342**	(0.033)	-0.246**	(0.057)
Cost*Scale	0.552**	(0.075)	0.156**	(0.066)
Standard deviation of random parameters				
None	3.553**	(0.138)	6.197**	(0.308)
Beach	1.515**	(0.063)	-2.982**	(0.166)
Coastal	0.004	(0.108)	0.607	(0.545)
International	-2.797**	(0.403)	5.354**	(0.895)
Foreign	0.633**	(0.123)	-2.874**	(0.452)
Csplit_25home	-0.247**	(0.019)	0.656**	(0.133)
Csplit_75home	0.561**	(0.028)	0.803**	(0.052)
Cost*Scale	1.580**	(0.113)	1.134**	(0.078)
Number of respondents	2,014		2,661	
Number of choices	10,070		13,305	
Log-Likelihood	-7,798		-10,184	
Adj. Rho-squared	0.291		0.299	
BIC	15,743		20,521	

Notes: 1,000 Sobol draws were used to simulate the likelihood; BIC: Bayesian information criterion; ** and * indicate the 1%- and 5%-level of significance, respectively. *None* is an alternative specific constant for choosing no programme, as opposed to a reduction programme. *Beach* and *coastal* represent the percentage reduction in the counties own beach and coastal waters (e.g., UK beaches for the UK treatment). The variable *International* and *Foreign* denote the percentage reduction in marine plastic pollution in international waters and the EEZ of the respective foreign partner country, respectively. *Csplit_25home* (*Csplit_75home*) is a dummy variable indicating that the respective home country incurs 25 percent (75 percent) of the overall programme costs compared to the reference of a 50 percent share. The variable Cost*Scale is the individual cost of the proposed programmes to the respondent in the choice experiment confounded with the scale of the multinomial logit model. Estimates of *Cost*Scale* are the mean and standard deviation of the normal distribution underlying the lognormal distribution assumed for this parameter with sign change.

Supplementary References

1. Meijer, L.J.J., van Emmerik, T., van der Ent, R., Schmidt, C. & Lebreton, L. More than 1000 rivers account for 80% of global riverine plastic emissions into the ocean. *Science Advances* 7, (2021).
2. Flanders Marine Institute. Maritime Boundaries Geodatabase: Maritime Boundaries and Exclusive Economic Zones (200NM). https://doi.org/10.14284/386 (2019).
3. Lebreton, L.C.M., van der Zwet, J., Damsteeg, J-W., Slat, B. Andrady, A. & Reisser, J. River plastic emissions to the world's oceans. *Nature Communications* 8, 15611 (2017).
4. Börger, T., Hanley, N., Johnston, R.J., Meginnis, K., Ndebele, T., Siyal, G.E.A., & de Vries, F. Equity preferences and abatement cost sharing in international environmental agreements. *American Journal of Agricultural Economics* 106(1), 416-441 (2024).
5. Train, K. & Weeks, M. Discrete Choice Models in Preference Space and Willingness-to-Pay Space. in *Applications of Simulation Methods in Environmental and Resource Economics* (eds. Scarpa, R. & Alberini, A.) 1-16 (Springer Netherlands, Dordrecht, 2005).
6. Train, K.E. *Discrete Choice Methods with Simulation*. (Cambridge University Press, Cambridge, 2009).
7. R Core Team. *R: A Language and Environment for Statistical Computing*. R Foundation for Statistical Computing (2017).
8. Hess, S. & Palma, D. Apollo: A flexible, powerful and customisable freeware package for choice model estimation and application. *Journal of Choice Modelling* 32, 100170 (2019).
9. Hess, S. & Palma, D. *Apollo: A Flexible, Powerful and Customisable Freeware Package for Choice Model Estimation and Application* – Version 0.2.4 – User Manual (2021).
10. GNI, PPP. https://data.worldbank.org/indicator/NY.GNP.MKTP.PP.CD?year_high_desc=true (2022).
